# Peanut AhmTERF1 Regulates Root Growth by Modulating Mitochondrial Abundance

**DOI:** 10.3390/genes14010209

**Published:** 2023-01-13

**Authors:** Limei Li, Xiaoyun Li, Chen Yang, Ling Li

**Affiliations:** 1 Guangdong Provincial Key Laboratory of Biotechnology for Plant Development, School of Life Science, South China Normal University, Guangzhou 510631, China; 2 School of Life Sciences, Zhaoqing University, Zhaoqing 526061, China

**Keywords:** mitochondria, peanut, root, *AhmTERF1*

## Abstract

Mitochondria are responsible for energy generation, as well as key metabolic and signaling pathways, and thus affect the entire developmental process of plants as well as their responses to stress. In metazoans, mitochondrial transcription termination factors (mTERFs) are known to regulate mitochondrial transcription. mTERFs have also been discovered in plants, but only a few of these proteins have been explored for their biological functions. Here, we report a role in root growth for mitochondria-associated protein *AhmTERF1* in peanut (*Arachis hypogaea* L.). Overexpressing *AhmTERF1* significantly stimulated the growth of peanut hairy roots and transgenic *Arabidopsis*. Surprisingly, *AhmTERF1* is predominantly expressed in the root meristem where it increases mitochondrial abundance. *AhmTERF1* binding to mtDNA was enriched in the *RRN18* and *RRN26* regions, suggesting it is related to the accumulation of mitochondrial ribosomes. Peanut is one of the main oil crops and the important source of edible oil and *AhmTERF1* likely affects agronomic traits related to root growth in different peanut cultivars. We propose that peanut *AhmTERF1* is an important protein for root growth due to its role in regulating mitochondrial abundance.

## 1. Introduction

Plant roots anchor the plant in the soil and are responsible for the uptake, storage and transport of minerals and water. They also communicate and interact with the soil microbiome and other plants, and sense biotic and abiotic stresses in the soil [1]. Overall, plants depend on root development, growth and function for their survival.

Mitochondria originate from endosymbiotic α-proteobacteria and possess their own vestigial genome, but most mitochondrial proteins are encoded by the nuclear genome and then are transported into the organelles. Mitochondria are essential for energy production [2], maintenance of calcium homeostasis [3] and the regulation of various intracellular signaling pathways; hence, they play crucial roles in plant growth, development and stress responses, such as seed germination [4,5], plant immunity [6] and the touch response [7,8].

A number of mitochondria-associated proteins that are involved in root growth and its regulation have been described. For example, *AtPHB3* is a conserved mitochondrial type I prohibitin, which is reported to maintain the mitochondrial morphology and cell division in the root meristem of *Arabidopsis* [9]. This finding also provides evidence of a role for mitochondria in regulating cell division in the root meristem. The *RETARDED ROOT GROWTH* (*RRG*) gene, encoding a mitochondrial protein, is predominantly expressed in the root meristem to control cell division [10]. *Arabidopsis slow growth3* (*slo3*) is a menber of a large family called pentatricopeptide repeat (PPR) proteins; when *slo3* mutates, the generation of meristem cells is hindered, resulting in reduced root apical meristem area [11]. These proteins all affect root growth and development by impacting cell division in the root meristem.

In view of the small size of and number of genes in their genomes, mitochondria seem to have an unexpectedly complex transcriptional regulatory network. The mitochondrial transcription termination factor (mTERF) proteins share repeated conserved sequences of 30-aa mTERF motif [12]. They were identified in human mitochondria three decades ago as important control factors of transcription termination [13]. In animals, only four mTERF members have been described [12], but there are more examples in plants, and they are predicted to be targeted to nucleus, chloroplasts, mitochondria or other subcellular locations [14], implying a range of complex and diverse functions. Nevertheless, in contrast to mammalian mTERFs, reports about the roles of plant mTERFs are still scarce. To date, these studies mostly focus on *Arabidopsis* and maize, which have 35 and 31 members of this mTERF family, respectively [12,15].

The first *mTERF* gene identified in higher plants, *SOLDAT10,* is localized to chloroplasts; in *Arabidopsis*, entirely inactivation of *SOLDAT10* lead to lethal mutation [16]. For other *Arabidopsis* mTERF mutants, including loss-of-function alleles of *BSM/RUG2* (*BELAYA SMERT/RUGOSA2*) [17,18], *MDA1* (*mTERF DEFECTIVE IN Arabidopsis1*) [19], *mTERF6* [20], *TWR-1/mTERF9* (*TWIRT1/mTERF9*) [21,22], *mTERF15* [23] and *SHOT1* (*SUPPRESSOR OF HOT1-4*) [24], stunted growth and altered organelle genes expression have been reported. Recently, molecular functions have been proposed for some of the *mTERF* genes characterised, all of which are related to post-transcriptional regulation of chloroplast and/or mitochondrial gene expression. BSM/RUG2 is necessary for the splicing of the second group II intron of the plastid *clpP* gene [17]. mTERF6 interacts with an RNA sequence in the chloroplast isoleucine transfer RNA gene (*trnI.2*) of the rRNA operon and promotes its maturation [20]. mTERF15 works in mitochondria as a splicing factor for *nad2* intron 3 splicing [23].

Peanut (*Arachis hypogaea* L.), an important economic and oil crop, possesses a complex allotetraploid genome due to its hybridization from two diploid species [25]. This complexity results in the existence of many genes, including the mTERF family, of unknown function. Our previous study indicated that, although the mTERF gene family underwent large changes as a result of the polyploidization event, some mTERFs were conserved. Among these, AhmTERF1 (Genebank of USA No. MG957109) is highly conserved between the cultivated allotetraploid species and its wild progenitors. Subcellular localization analysis indicates that the AhmTERF1 protein is closely associated with mitochondria [26], consistent with it having a mitochondrial function.

Here, we report the function of AhmTERF1. *AhmTERF1* is predominantly expressed in the root meristem. In consideration of its subcellular localization, we further figure out the relationship of AhmTERF1 with mitochondria and the regulation for peanut root growth by modulating mitochondrial abundance. In conclusion, our results reveal an unexpected role of AhmTERF1 and highlight the importance of this protein in peanut development.

## 2. Materials and Methods

### 2.1. Vector Construction

For the *pAhmTERF1::GUS* fusion construct, a 1.531-kb upstream sequence from the *AhmTERF1* gene was amplified from peanut genomic DNA using the primers shown in Supplementary Table S1. The PCR product was then cloned into the HY107 vector [27]. To construct the *35S::AhmTERF1-GFP* and *35S::AhmTERF1-mcherry* plasmids, the *AhmTERF1* coding sequence was amplified from peanut cDNA (for the primers used, see Supplementary Table S1). The PCR products were then cloned into the *35S::GFP* vector or the *35S::mcherry* vector. The *AhmTERF1 RNAi* construct was made by associate research fellow Xu Liu of South China Botanical Garden, Chinese Academy of Sciences according to the method [28].

### 2.2. Plant Materials and Growth Conditions

Peanut was grown in pots with a mixed soil consisting of vermiculite, perlite and soil (1:1:1), and cultivated at the condition of 28 °C and 16 h light photoperiod for 3 days. The *35S::eGFP*, *35S::AhmTERF1-GFP*, *35S::AhmTERF1 RNAi* and *pAhmTERF1::GUS* constructs were transformed into *Agrobacterium rhizogenes* K599 to induce hairy roots, according to the protocol [29].

*Arabidopsis* plants were grown in potting soil with 16 h light/8 h dark photoperiod. To obtain *AhmTERF1* overexpression and *AhmTERF1::GUS Arabidopsis* plants, constructs were transformed into the Col-0 strain of *Arabidopsis* by the floral dip method as previous reported [30]. *AhmTERF1* overexpressing *Arabidopsis* plants were selected on 1/2 MS medium containing 50 mg/L kanamycin. *AhmTERF1::GUS Arabidopsis* plants were selected using the herbicide.

### 2.3. Quantitative Real-Time PCR (qRT-PCR)

RNA was extracted from peanut roots as described by Wan and Li [31]. Reverse transcription process was carried out using the HiScript^®^ III 1st Strand cDNA Synthesis Kit (+gDNA wiper) (Vazyme). ChamQ Universal SYBR qPCR Master Mix (Vazyme) was used according to the manufacturer’s instructions with a BioRad CFX96 Real-Time PCR detection system. Primers for qRT-PCR are listed in Supplementary Table S1.

### 2.4. Histochemical Staining of GUS Activity

According to a previous reported methodology [32], histochemical staining of GUS activity was carried out. To remove chlorophyll, stained samples were soaked with 70% ethanol for 24 h. Nomarski DIC images of GUS expression were taken as mentioned in Xu and Scheres [32].

### 2.5. Subcellular Localization Analysis

To visualise fluorescent fusion proteins, leaves of 2-week old Col-0 *Arabidopsis* grown on soil were incubated for 3 h at 26 °C in the dark in a protoplasting solution (1.5% cellulase, 0.75% macerozyme, 0.5  M mannitol, 10  mM MES pH 5.7, 10  mM CaCl_2_ and 0.1% BSA). The isolation and transformation of protoplasts were performed as described [33], and then protoplasts were observed with a confocal laser scanning microscopy (LSM800, Carl Zeiss, Germany).

### 2.6. Western Blotting

According to the reported protocol [5] with minor modifications, the extraction of a crude preparation of mitochondria was conducted. Approximately 10 g of young roots was ground at 4 °C in extraction buffer (10 mM KH_2_PO_4_, pH 7.5, 0.3 M sucrose, 2 mM EDTA, 5 mM tetrasodium pyrophosphate, 5 mM cysteine, 20 mM ascorbic acid, 1% polyvinylpyrrolidone 40, 1% BSA and 10% glycerin). Two layers of Miracloth were used for filtering. And then the homogenate was centrifuged in a process of 5 min at 3000× *g*. Mitochondria were extracted from the clear supernatant by centrifugation at 20,000× *g* for 10 min. The AhmTERF1 antibody was made by Willget Biotech Co., Ltd. (Shanghai, China). Western blotting was conducted as described previously [33].

### 2.7. Ultrastructural Analyses

The ultrastructural analyses were carried out according to the method previous reported [34]. 2.5% glutaraldehyde solution was used to fix the hairy root tips at 4 °C for 12 h. Then the glutaraldehyde solution was discarded and phosphate-buffered saline (PBS; 0.1 M, pH 7.0) was added and allowed to stand for 15 min. The hairy root tips were washed three times with PBS after which 1% osmic acid was added and the roots were soaked for 2 h. The osmic acid was discarded and PBS was added and allowed to stand for another 15 min. Hairy root tips were washed three times with PBS and then the hairy root tips were dehydrated with alcohol step by step (30%→50%→70%→80%→90%→100%; 15 min at each concentration). Then the roots were treated with 100% alcohol for 20 min, and finally with acetone for 20 min.

The roots were submerged in osmotic solution Ⅰ (Spurr embedding agent mixed with acetone (*v*/*v* = 1/1)) for 1 h, then removed to osmotic solution Ⅱ (Spurr embedding agent mixed with acetone (*v*/*v* = 3/1)) for 3 h. The samples were then transferred to Spurr embedding agent overnight. The permeated samples were then heated overnight in a 70 °C oven to complete the embedding treatment. The samples were processed using a Leica EM UC7 ultrathin slicer to obtain 70–90 nm ultrathin slices, which were imaged using a Hitachi H-7650 transmission electron microscope.

### 2.8. ChIP-qPCR Assay

The ChIP assay was conducted as previous described [35]. Primers for qPCR are listed in Supplementary Table S1. An intergenic region (atp9) that does not bind AhmTERF1 was used as a negative control.

### 2.9. mtDNA Copy Number Analysis

The mtDNA copy number was determined by reference to Mondal et al. [36] and Mei et al. [37]. The relative mtDNA copy number is defined as the ratio of mtDNA to nuclear DNA. mtDNA was labeled with RRN18 and RRN26, and nuclear DNA was labeled with a single-copy gene, Arahy.U6ZXMA.

### 2.10. Statistical Analysis

Results are expressed as mean values ± standard deviation (SD). Data were evaluated by one-way analysis of variance (ANOVA) or the Student t-test using SPSS19.0 software. *p* < 0.05 were considered as significance.

## 3. Results

### 3.1. Overexpression of AhmTERF1 Promotes Root System Architecture

**Figure 1:**
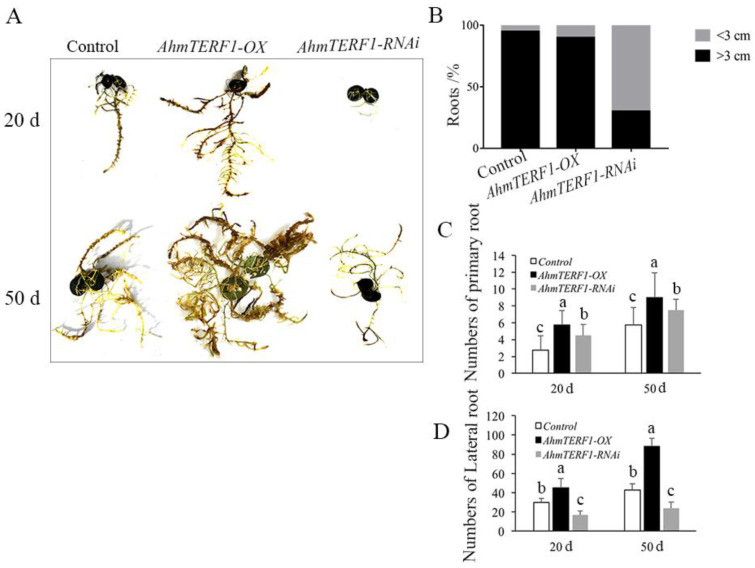
*AhmTERF1* affects the growth of peanut hairy roots. (**A**): Phenotype of 20 d-old and 50 d-old peanut hairy roots in control, *AhmTERF1*-overexpression (*AhmTERF1-OX*) and *AhmTERF1*-silenced (*AhmTERF1-RNAi*) lines. (**B**): Proportion of roots of different length, i.e., <3 cm or >3 cm. (**C**,**D**): Number of primary roots and lateral roots in 20 d-old and 50 d-old seedlings. Lower case letters (a, b, c) indicate significantly different groups (*p* < 0.05).

To investigate the function of *AhmTERF1*, we first transformed peanut hairy roots with a vector carrying either a fusion of the CaMV35S promoter and *AhmTERF1* cDNA or a *AhmTERF1* RNAi construct to obtain *AhmTERF1* overexpression (*AhmTERF1-OX*) and *AhmTERF1*-silenced hairy root lines. It was found that the number of primary and lateral roots increased in *AhmTERF1-OX* lines. However, 70% of *AhmTERF1*-RNAi hairy roots were thinner and shorter (<3 cm) and exhibited growth retardation (Figure 1). This suggests a role for *AhmTERF1* in hairy root formation in peanut. In other words, hairy roots were stimulated to develop when *AhmTERF1* was overexpressed, but roots were stunted when *AhmTERF1* expression was silenced. We obtained similar results in transgenic *Arabidopsis* lines overexpressing *AhmTERF1*: two *AhmTERF1* overexpression lines with increased root length and number are shown in Figure S1.

### 3.2. AhmTERF1 Is Preferentially Expressed in the Root Meristem

**Figure 2:**
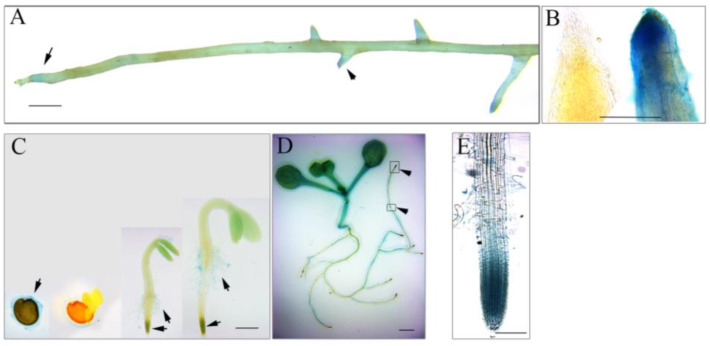
Tissue distribution of *AhmTERF1* gene expression. (**A**): GUS staining of *pAhmTERF1:GUS* hairy roots. Scale bar: 0.5 cm. (**B**): Root tips of hairy roots (Left: Control hariy root; Right: *pAhmTERF1:GUS* hairy roots). Scale bar: 200 µm. (**C**): GUS staining of transgenic *pAhmTERF1:GUS Arabidopsis* seeds and seedlings (images from left): seed imbibing water; seed at germination stage; seedlings 3 and 5 d after the onset of germination, respectively. Scale bar: 0.5 cm. (**D**): GUS staining of transgenic *pAhmTERF1:GUS Arabidopsis* seedling 7 d after the onset of germination. The boxes and the arrowheads indicate root tips. Scale bar: 0.5 cm. (**E**): Larger version of root tip. Scale bar: 100 µm.

**Figure 3:**
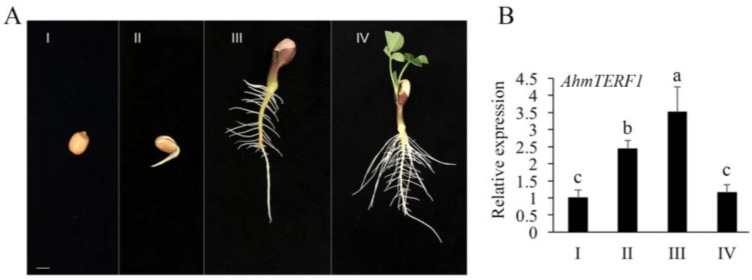
Expression pattern of *AhmTERF1* at various peanut root growth stages. (**A**): Phenotype at various peanut root growth stages. Scale bar: 1 cm. (**B**): *AhmTERF1* expression level at the corresponding root growth stages. Lower case letters (a, b, c) indicate significantly different groups (*p* < 0.05).

To understand the role of *AhmTERF1* in root system architecture, we fused the *AhmTERF1* promoter to the GUS reporter gene and transformed the construct into peanut hairy roots and Col *Arabidopsis*. The hairy roots containing *pAhmTERF1:GUS* displayed a GUS expression pattern in the primary and lateral root tips (Figure 2A,B). *Arabidopsis* transgenic plants showed GUS expression signal in the mucilage of imbibed seeds, root hairs and seedling stems, and especially in the meristematic zone of primary and lateral root tips (Figure 2C–E), consistent with the results obtained with transgenic peanut hairy roots. To further explore *AhmTERF1* expression, we next investigated the expression pattern of *AhmTERF1* in peanut roots at different development stages. Q-PCR results showed that *AhmTERF1* was upregulated as peanut roots develop (Figure 3), indicating that *AhmTERF1* is involved in the root system architecture of peanut.

### 3.3. AhmTERF1 Overexpression Increases the Number of Mitochondria in Hairy Roots

**Figure 4:**
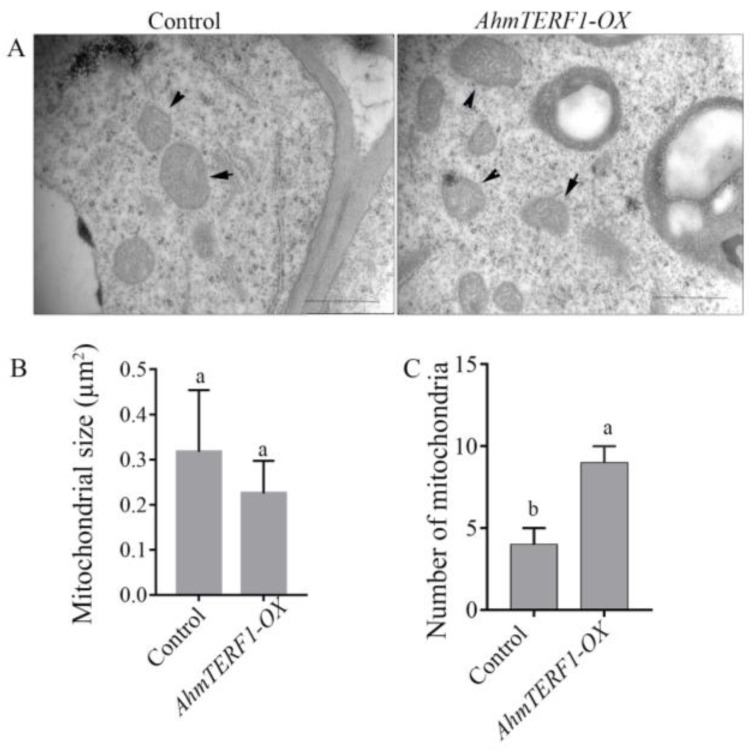
Mitochondria in the tips of Control and *AhmTERF1-OX* hairy roots shown by transmission electron microscopy. (**A**): Images of mitochondria in the root tip of 20 d-old Control and *AhmTERF1-OX* hairy roots. Scale bars: 1 µm. (**B**,**C**): The size and number of mitochondria in the root tips of 20 d-old Control and *AhmTERF1-OX* hairy roots. Lower case letters (a, b) indicate significantly different groups (*p* < 0.05).

*AhmTERF1* has been localized within cells to a position close to mitochondria [26]. To further confirm the role of *AhmTERF1* in mitochondria and its function in root growth, we investigated the size and number of mitochondria in *AhmTERF1-OX* hairy root tips by transmission electron microscopy. The size and morphology of mitochondria in *AhmTERF1-OX* hairy roots were similar to those of controls. However, the number of mitochondria was significantly higher in *AhmTERF1-OX* hairy roots (Figure 4).

**Figure 5:**
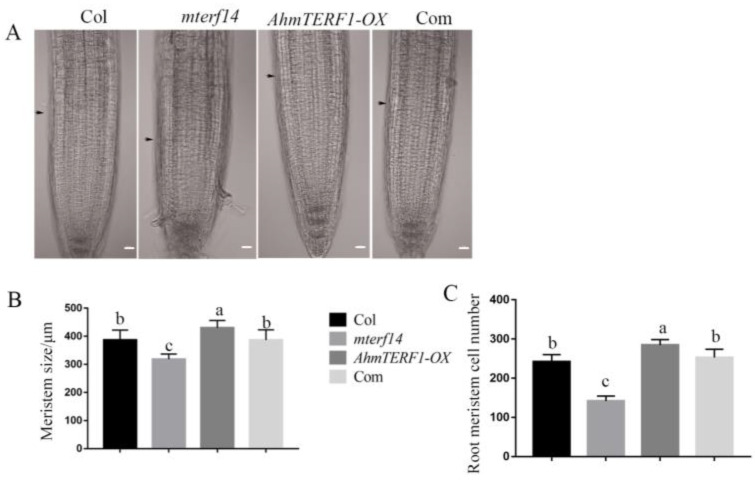
*AhmTERF1* accelerates root meristem growth in *Arabidopsis* plants. (**A**): Images of root meristem in *AhmTERF1-OX* transgenic *Arabidopsis*. Scale bars: 20 µm. Arrows indicate the boundary between the root meristem and the elongation zone. (**B**,**C**): Meristem size and cell number of roots in A. Com indicates *35S:AhmTERF1/mterf14 Arabidopsis* plants. Lower case letters (a, b, c) indicate significantly different groups (*p* < 0.05).

Mitochondrial defects in the *Arabidopsis mterf14* mutant result in a dwarf phenotype. Therefore, we hypothesized that *AhmTERF1* might restore the dwarf phenotype of the *mterf14* mutant. Indeed, *AhmTERF1mterf14* plants exhibited a normal Col phenotype, confirming our hypothesis (Supplemental Figure S2). By examining the root tips, we showed that the size of the meristem region and the number of cells decreased in the *mterf14* mutant, but were higher than controls in the *AhmTERF1-OX* line. In *35S:AhmTERF1/mterf14 Arabidopsis* plants, the short-root phenotype of the *mterf14* mutant was restored to that of controls (Figure 5), implying that *AhmTERF1* regulates root growth by promoting the activity of the root tip meristem.

**Figure 6:**
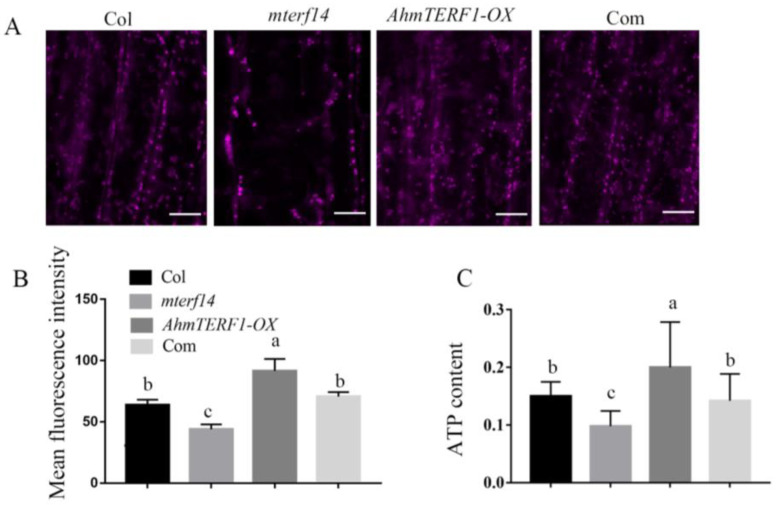
Mitochondria and ATP content are increased in *AhmTERF1-OX Arabidopsis*. (**A**): Images of mitochondria in the root tip of 3-d old *Arabidopsis* taken by confocal laser scanning microscope (LSM800, Carl Zeiss, Germany). Scale bars: 5 µm. MitoTracker was used as a mitochondrial marker. (**B**): Fluorescence intensity of A. (**C**): ATP content of the roots of 3-d old *Arabidopsis* seedlings. ATP content was calculated as µmol/g. Com indicates *35S:AhmTERF1/mterf14 Arabidopsis* plants. Lower case letters (a, b, c) indicate significantly different groups (*p* < 0.05).

We next treated the root tip with MitoTracker Red CMXRos to fluorescently mark mitochondria. Compared with Col and *mterf14* mutant plants, the intensity of fluorescence in *AhmTERF1-OX* root tips was higher, which is consistent with an elevated number of mitochondria in *AhmTERF1-OX* plants (Figure 6A,B). ATP content correlated with fluorescence levels (Figure 6C). Overall, these results suggest that *AhmTERF1* is crucial for facilitating mitochondrial accumulation.

### 3.4. Identification of the Target Genes of AhmTERF1

**Figure 7:**
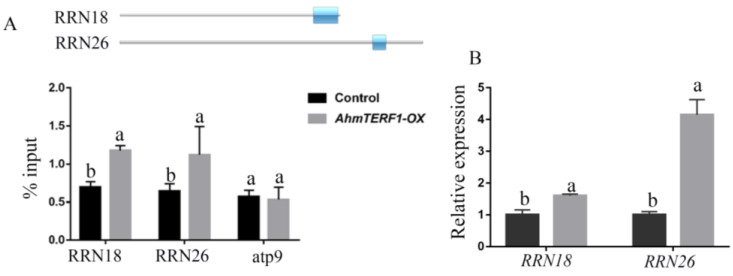
AhmTERF1 binds specifically to and regulates its target rRNA genes, RRN18 and RRN26. (**A**): Enrichment of AhmTERF1 binding to RRN18 and RRN26 DNA sequences shown by ChIP-qPCR, with atp9 as negative control. Cox is used as an internal reference. The upper figure represents gene sequences. Blue rectangles represent the enrichment region. (**B**): *RRN18* and *RRN26* expression in Control and *AhmTERF1-OX* hairy roots. Lower case letters (a, b) indicate significantly different groups (*p* < 0.05).

We supposed that the target genes of AhmTERF1 would be located in the mitochondrial genome. ChIP-qPCR indicated that AhmTERF1 was enriched in regions of the *RRN18* and *RRN26* rRNA genes (Figure 7A). Furthermore, Q-PCR results suggested that the expression levels of *RRN18* and *RRN26* were enhanced in *AhmTERF1-OX* hairy roots (Figure 7B). 18S RNA and 26S RNA, the products of *RRN18* and *RRN26*, are important components of mitochondrial ribosomes and are involved in their assembly. Presumably, AhmTERF1 promotes *RRN18* and *RRN26* expression to affect accumulation of mitochondrial ribosomes, and thus to influence the number of mitochondria in cells.

### 3.5. AhmTERF1 Expression in Different Cultivars of Peanut

**Figure 8:**
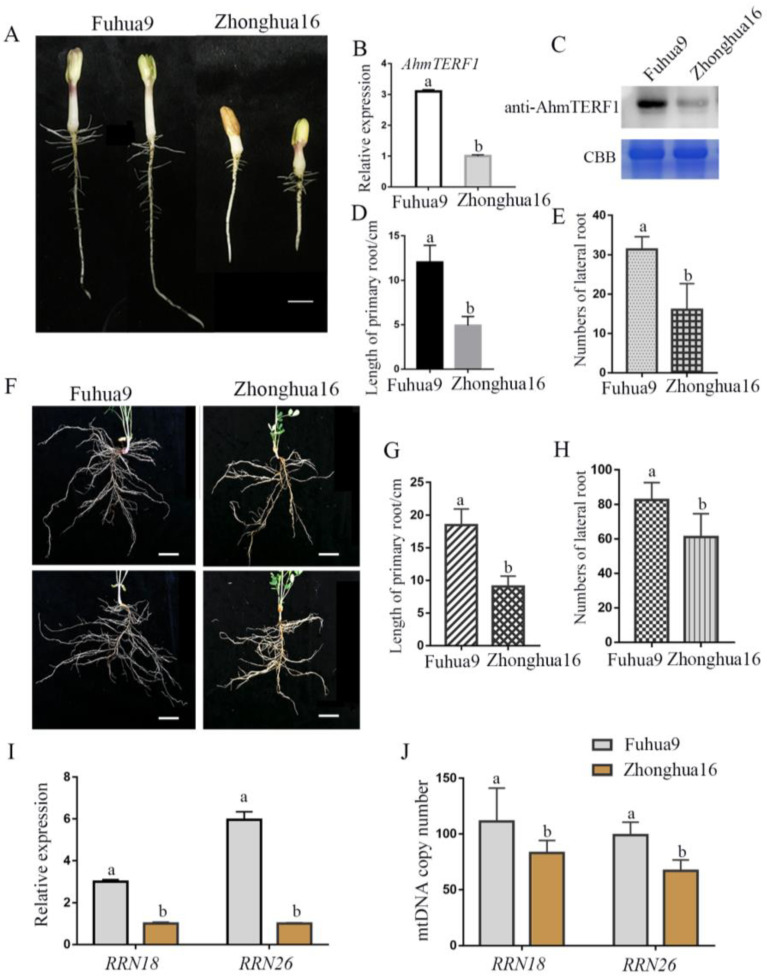
The effect of *AhmTERF1* expression on root growth and mtDNA copy number in Fuhua9 and Zhonghua16 peanut cultivars. (**A**): Phenotype of 5 d-old Fuhua9 and Zhonghua16 cultivars. (**B**,**C**): AhmTERF1 transcript and protein expression, respectively, in the roots of 5 d-old Fuhua9 and Zhonghua16. (**D**,**E**): Length of primary root and number of lateral roots of 5 d-old Fuhua9 and Zhonghua16. (**F**): Phenotype of 25 d-old Fuhua9 and Zhonghua16 cultivars. (**G**,**H**): Length of primary root and number of lateral roots, respectively, of 25 d-old Fuhua9 and Zhonghua16. (**I**): *RRN18* and *RRN26* expression in the roots of 5 d-old Fuhua9 and Zhonghua16. (**J**): mtDNA copy number in the roots of 5 d-old Fuhua9 and Zhonghua16. mtDNA was labeled with RRN18 and RRN26. Lower case letters (a, b) indicate significantly different groups (*p* < 0.05).

Different cultivars of peanut exhibit different agronomic traits, for example, varying in the characteristics of their roots. Fuhua9, which has a well-developed root system, showed stronger expression of AhmTERF1 than Zhonghua16, which has a relatively underdeveloped root system at the same stage (5 d) of seedling development (Figure 8A–H). The target genes of AhmTERF1, *RRN18* and *RRN26*, were also variably expressed in these two peanut cultivars. Thus, *RRN18* and *RRN26* showed higher transcript levels in Fuhua9 than in Zhonghua16 (Figure 8I). We also measured the abundance of mitochondrial DNA relative to a nuclear gene to evaluate mtDNA copy number. In Fuhua9, the mtDNA copy number was higher than in Zhonghua16 (Figure 8J). These results indicate a role for AhmTERF1 in regulating agronomic traits. In particular, it seems likely that those peanut cultivars with strong AhmTERF1 expression have a more extensive root system.

### 3.6. Mechanism of AhmTERF1 to Regulate Root Growth

**Figure 9:**
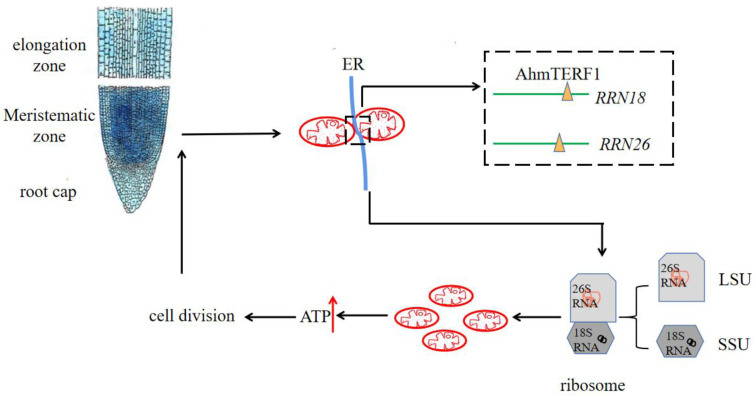
Working model of AhmTERF1 function.

In order to determine accurately the location of AhmTERF1 within cells, we futher conducted the subcellular localization assay and it is revealed the co-localization of AhmTERF1 with the ER and mtDNA (Supplemental Figure S3). To sum up, we can speculate that AhmTERF1 is located at ER-mitochondrion contract sites to regulate root growth by modulating mitochondrial abundance (Figure 9).

AhmTERF1 is preferentially expressed in the root meristem. At mitochondrion-ER contact sites, AhmTERF1 combines with mtDNA, and is enriched in the RRN18 and RRN26 loci. The respective transcripts 18S RNA and 26S RNA are involved in the assembly of mitochondrial ribosomes, which promotes an increase in the number of mitochondria. Thus, energy production is increased and can then be used for cell division, which will increase the extent of the meristematic zone and result in peanut root growth.

## 4. Discussion

The mitochondrion is one of the most malleable organelles in the cell. It can change its abundance, structure and distribution in the cytoplasm according to specific energy requirements. In plants, when levels of energy demand and metabolic flux are high, this is reflected in an increase in the number or area of mitochondria, as well as in higher levels of mitochondrial electron transport chain (mETC) components [38,39,40]. During the seed germination, development of pollen and ripening of fruit stages, mitochondria show the highest respiration rate [41]. Pollen respiration increases 10-fold, and the number of mitochondria increases 20 to 40 times, in meiotic and tapetal cells [38]. The doubling of mitochondrial volume and number, as well as a boost in mitochondrial dynamics, occur in germinated seedlings to provide energy for seedling growth and development [4]. In the root apical meristem of *Arabidopsis*, along with an increasing of respiratory rate, an approximately 3-fold increase in mitochondrial number have been found [41]. Within the root meristem, cell division is important to maintain the root meristem activity and support root growth [42]. Thus, presumably, our observations of an increase in the number of mitochondria in peanut root tips indicate an acceleration in respiratory activity, providing energy for root growth.

In comparison with other organelles, biosynthesis displays a particular level of complexity in mitochondria, because they contain their own genomes and specialized ribosomes. The mitochondrial translation process seems to be a key point to monitor mitochondrial homeostasis and may play a part in establishing the abundance of mitochondria within the cells.

Previous research has shown that mTERFs are involved in ribosome biogenesis. Lack of DmMTERF3 was found in Drosophila to be related with a reducing of the amount of 16S rRNA and the assembly of mitochondrial ribosomes was hindered [43]. MTERF4 and NSUN4 RNA methyltransferase join together to form a complex, which is necessary to target NSUN4 to the mammalian mitochondrial ribosome [44]. We found that binding of AhmTERF1 to mtDNA was enriched in the region containing the rRNA genes, *RRN18* and *RRN26*. 18S RNA and 26S RNA, the products of these genes, are essential components of mitochondrial ribosomes and are crucial for their assembly. In the mitochondrial genome of higher plants, three ribosomal RNA (rRNA) genes, including *rrn26*, *rrn18* and *rrn5*, encode 26S, 18S and 5S rRNAs, respectively [45]. Post-transcriptional modifications of rRNAs are essential for ribosome biogenesis and translation [46]. In spite of their greater diversity in plants, knowledge of mTERF functions in mitochondrial ribosomal biogenesis remains sparse. For example, levels of 16S and 23S rRNAs were decreased in *Zm-mterf4* mutants of maize, showing the important role of *Zmmterf4* in the accumulation of plastid ribosomes [47]. In this paper, we speculate that the expression of *RRN18* and *RRN26* accelerates the accumulation of mitochondrial ribosomes, thereby increasing the number of mitochondria and supporting the growth of roots.

In addition, this paper revealed the co-localization of AhmTERF1 with the ER and mtDNA (Supplemental Figure S3). Mitochondria have a close relationship with the ER; ER-mitochondrion contact sites is important for mitochondrial fission, lipid transfer, calcium signaling [48] and the synchronization of mtDNA synthesis [49]. The majority (84%) of mitochondrial fission events happen at this location [50]. The unit of mtDNA inheritance, nucleoids contain lots of copies of mtDNA and display distribution in mitochondrial networks. In mammalian cells, these nucleoids linked to a small subset of ER-mitochondrion contact points spatially and temporally, involve in mtDNA synthesis. Before mitochondrial constriction and division. mtDNA replication occurs. ER-mitochondrion contact sites regulate mtDNA replication and distribute the newly replicated mtDNA to progeny mitochondria [49]. In our previous study, it was found that AhmTERF1 was closely surrounded by mitochondria [26]. This might suggest that AhmTERF1 is located at ER-mitochondrion contract sites and that the mitochondria around AhmTERF1 are daughter mitochondria produced by mitochondrial division at those sites (Figure 9).

## Data Availability

The data that support the findings of this study are available from the authors, upon reasonable request.

## Supplementary Materials

The following supporting information can be downloaded at: https://www.mdpi.com/article/10.3390/genes14010209/s1, Figure S1: *AhmTERF1* promotes root growth in *Arabidopsis*; Figure S2: *AhmTERF1* restores the phenotype of *mterf14* in *Arabidopsis*; Figure S3: AhmTERF1 co-localizes with mtDNA and ER; Table S1: Primers used in this study.

## References

[1] Karlova R., Boer D., Hayes S., Testerink C. (2021). Root plasticity under abiotic stress. Plant Physiol..

[2] Snyder C., Stefano G.B. (2015). Mitochondria and chloroplasts shared in animal and plant tissues: Significance of communication. Med. Sci. Monit..

[3] Sarasija S., Norman K.R. (2015). A γ-Secretase Independent Role for Presenilin in Calcium Homeostasis Impacts Mitochondrial Function and Morphology in Caenorhabditis elegans. Genetics.

[4] Paszkiewicz G., Gualberto J.M., Benamar A., Macherel D., Logan D.C. (2017). *Arabidopsis* Seed Mitochondria Are Bioenergetically Active Immediately upon Imbibition and Specialize via Biogenesis in Preparation for Autotrophic Growth. Plant Cell.

[5] Ma W., Guan X., Li J., Pan R., Wang L., Liu F., Ma H., Zhu S., Hu J., Ruan Y.L. (2019). Mitochondrial small heat shock protein mediates seed germination via thermal sensing. Proc. Natl. Acad. Sci. USA.

[6] Huang Y., Chen X., Liu Y., Roth C., Copeland C., McFarlane H.E., Huang S., Lipka V., Wiermer M., Li X. (2013). Mitochondrial AtPAM16 is required for plant survival and the negative regulation of plant immunity. Nat. Commun..

[7] Xu Y., Berkowitz O., Narsai R., De Clercq I., Hooi M., Bulone V., Van Breusegem F., Whelan J., Wang Y. (2019). Mitochondrial function modulates touch signalling in *Arabidopsis* thaliana. Plant J..

[8] Fernie A.R. (2019). Making sense of the complex role of the mitochondria in mediating the plant touch response. Plant J..

[9] Van Aken O., Pecenková T., van de Cotte B., De Rycke R., Eeckhout D., Fromm H., De Jaeger G., Witters E., Beemster G.T., Inzé D. (2007). Mitochondrial type-I prohibitins of *Arabidopsis* thaliana are required for supporting proficient meristem development. Plant J..

[10] Zhou X., Li Q., Chen X., Liu J., Zhang Q., Liu Y., Liu K., Xu J. (2011). The *Arabidopsis* RETARDED ROOT GROWTH gene encodes a mitochondria-localized protein that is required for cell division in the root meristem. Plant Physiol..

[11] Hsieh W.Y., Liao J.C., Hsieh M.H. (2015). Dysfunctional mitochondria regulate the size of root apical meristem and leaf development in *Arabidopsis*. Plant Signal. Behav..

[12] Kleine T., Leister D. (2015). Emerging functions of mammalian and plant mTERFs. Biochim. Biophys. Acta.

[13] Kruse B., Narasimhan N., Attardi G. (1989). Termination of transcription in human mitochondria: Identification and purification of a DNA binding protein factor that promotes termination. Cell.

[14] Kleine T. (2012). *Arabidopsis* thaliana mTERF proteins: Evolution and functional classification. Front. Plant Sci..

[15] Zhao Y., Cai M., Zhang X., Li Y., Zhang J., Zhao H., Kong F., Zheng Y., Qiu F. (2014). Genome-wide identification, evolution and expression analysis of mTERF gene family in maize. PLoS ONE.

[16] Meskauskiene R., Würsch M., Laloi C., Vidi P.A., Coll N.S., Kessler F., Baruah A., Kim C., Apel K. (2009). A mutation in the *Arabidopsis* mTERF-related plastid protein SOLDAT10 activates retrograde signaling and suppresses (1)O(2)-induced cell death. Plant J..

[17] Babiychuk E., Vandepoele K., Wissing J., Garcia-Diaz M., De Rycke R., Akbari H., Joubès J., Beeckman T., Jänsch L., Frentzen M. (2011). Plastid gene expression and plant development require a plastidic protein of the mitochondrial transcription termination factor family. Proc. Natl. Acad. Sci. USA.

[18] Quesada V., Sarmiento-Mañús R., González-Bayón R., Hricová A., Pérez-Marcos R., Graciá-Martínez E., Medina-Ruiz L., Leyva-Díaz E., Ponce M.R., Micol J.L. (2011). *Arabidopsis* RUGOSA2 encodes an mTERF family member required for mitochondrion, chloroplast and leaf development. Plant J..

[19] Robles P., Micol J.L., Quesada V. (2012). *Arabidopsis* MDA1, a nuclear-encoded protein, functions in chloroplast development and abiotic stress responses. PLoS ONE.

[20] Romani I., Manavski N., Morosetti A., Tadini L., Maier S., Kühn K., Ruwe H., Schmitz-Linneweber C., Wanner G., Leister D. (2015). A Member of the *Arabidopsis* Mitochondrial Transcription Termination Factor Family Is Required for Maturation of Chloroplast Transfer RNAIle(GAU). Plant Physiol..

[21] Mokry M., Nijman I.J., van Dijken A., Benjamins R., Heidstra R., Scheres B., Cuppen E. (2011). Identification of factors required for meristem function in *Arabidopsis* using a novel next generation sequencing fast forward genetics approach. BMC Genom..

[22] Robles P., Micol J.L., Quesada V. (2015). Mutations in the plant-conserved MTERF9 alter chloroplast gene expression, development and tolerance to abiotic stress in *Arabidopsis* thaliana. Physiol. Plant.

[23] Hsu Y.W., Wang H.J., Hsieh M.H., Hsieh H.L., Jauh G.Y. (2014). *Arabidopsis* mTERF15 is required for mitochondrial nad2 intron 3 splicing and functional complex I activity. PLoS ONE.

[24] Kim M., Lee U., Small I., des Francs-Small C.C., Vierling E. (2012). Mutations in an *Arabidopsis* mitochondrial transcription termination factor-related protein enhance thermotolerance in the absence of the major molecular chaperone HSP101. Plant Cell.

[25] Zhuang W., Chen H., Yang M., Wang J., Pandey M.K., Zhang C., Chang W.C., Zhang L., Zhang X., Tang R. (2019). The genome of cultivated peanut provides insight into legume karyotypes, polyploid evolution and crop domestication. Nat. Genet..

[26] Li L.M., Hu B., Li X.Y., Li L. (2020). Characterization of mTERF family in allotetraploid peanut and their expression levels in response to dehydration stress. Biotechnol. Biotec. Eq..

[27] Zhou Y., Zeng L., Hou X., Liao Y., Yang Z. (2020). Low temperature synergistically promotes wounding-induced indole accumulation by INDUCER OF CBF EXPRESSION-mediated alterations of jasmonic acid signaling in Camellia sinensis. J. Exp. Bot..

[28] Qian Q., Yang Y., Zhang W., Hu Y., Li Y., Yu H., Hou X. (2021). A novel *Arabidopsis* gene RGAT1 is required for GA-mediated tapetum and pollen development. New Phytol..

[29] Liu S., Su L., Liu S., Zeng X., Zheng D., Hong L., Li L. (2016). Agrobacteriumrhizogenes-mediated transformation of Arachis hypogaea: An efficient tool for functional study of genes. Biotechnol. Biotec. Eq..

[30] Clough S.J., Bent A.F. (1998). Floral dip: A simplified method for Agrobacterium-mediated transformation of *Arabidopsis* thaliana. Plant J..

[31] Wan X., Li L. (2005). Molecular cloning and characterization of a dehydration-inducible cDNA encoding a putative 9-cis-epoxycarotenoid dioxygenase in *Arachis hygogaea* L.. DNA Seq..

[32] Xu J., Scheres B. (2005). Dissection of *Arabidopsis* ADP-RIBOSYLATION FACTOR 1 function in epidermal cell polarity. Plant Cell.

[33] Liu X., Li L., Li M., Su L., Lian S., Zhang B., Li X., Ge K., Li L. (2018). AhGLK1 affects chlorophyll biosynthesis and photosynthesis in peanut leaves during recovery from drought. Sci. Rep..

[34] Fang L., Hou X., Lee L.Y., Liu L., Yan X., Yu H. (2011). AtPV42a and AtPV42b redundantly regulate reproductive development in *Arabidopsis* thaliana. PLoS ONE.

[35] Qiu Q., Mei H., Deng X., He K., Wu B., Yao Q., Zhang J., Lu F., Ma J., Cao X. (2019). DNA methylation repels targeting of *Arabidopsis* REF6. Nat. Commun..

[36] Mondal R., Ghosh S.K., Choudhury J.H., Seram A., Sinha K., Hussain M., Laskar R.S., Rabha B., Dey P., Ganguli S. (2013). Mitochondrial DNA copy number and risk of oral cancer: A report from Northeast India. PLoS ONE.

[37] Mei H., Sun S., Bai Y., Chen Y., Chai R., Li H. (2015). Reduced mtDNA copy number increases the sensitivity of tumor cells to chemotherapeutic drugs. Cell Death Dis..

[38] Warmke H.E., Lee S.L. (1978). Pollen Abortion in T Cytoplasmic Male-Sterile Corn (Zea mays): A Suggested Mechanism. Science.

[39] Huang J., Struck F., Matzinger D.F., Levings C.S. (1994). Flower-enhanced expression of a nuclear-encoded mitochondrial respiratory protein is associated with changes in mitochondrion number. Plant Cell.

[40] Zabaleta E., Heiser V., Grohmann L., Brennicke A. (1998). Promoters of nuclear-encoded respiratory chain complex I genes from *Arabidopsis* thaliana contain a region essential for anther/pollen-specific expression. Plant J..

[41] Liberatore K.L., Dukowic-Schulze S., Miller M.E., Chen C., Kianian S.F. (2016). The role of mitochondria in plant development and stress tolerance. Free Radic. Biol. Med..

[42] Perilli S., Di Mambro R., Sabatini S. (2012). Growth and development of the root apical meristem. Curr. Opin. Plant Biol..

[43] Wredenberg A., Lagouge M., Bratic A., Metodiev M.D., Spåhr H., Mourier A., Freyer C., Ruzzenente B., Tain L., Grönke S. (2013). MTERF3 regulates mitochondrial ribosome biogenesis in invertebrates and mammals. PLoS Genet..

[44] Cámara Y., Asin-Cayuela J., Park C.B., Metodiev M.D., Shi Y., Ruzzenente B., Kukat C., Habermann B., Wibom R., Hultenby K. (2011). MTERF4 regulates translation by targeting the methyltransferase NSUN4 to the mammalian mitochondrial ribosome. Cell Metab..

[45] Petersen G., Cuenca A., Møller I.M., Seberg O. (2015). Massive gene loss in mistletoe (Viscum, Viscaceae) mitochondria. Sci. Rep..

[46] Van Haute L., Hendrick A.G., D’Souza A.R., Powell C.A., Rebelo-Guiomar P., Harbour M.E., Ding S., Fearnley I.M., Andrews B., Minczuk M. (2019). METTL15 introduces N4-methylcytidine into human mitochondrial 12S rRNA and is required for mitoribosome biogenesis. Nucleic Acids Res..

[47] Hammani K., Barkan A. (2014). An mTERF domain protein functions in group II intron splicing in maize chloroplasts. Nucleic Acids Res..

[48] Friedman J.R., Voeltz G.K. (2011). The ER in 3D: A multifunctional dynamic membrane network. Trends Cell Biol..

[49] Lewis S.C., Uchiyama L.F., Nunnari J. (2016). ER-mitochondria contacts couple mtDNA synthesis with mitochondrial division in human cells. Science.

[50] Guo Y., Li D., Zhang S., Yang Y., Liu J.J., Wang X., Liu C., Milkie D.E., Moore R.P., Tulu U.S. (2018). Visualizing Intracellular Organelle and Cytoskeletal Interactions at Nanoscale Resolution on Millisecond Timescales. Cell.

