# Supplementary material for: Peanut AhmTERF1 Regulates Root Growth by Modulating Mitochondrial Abundance
Source: Genes (Basel). 2023 Jan 13;14(1):209. doi: 10.3390/genes14010209 (PMC9859088; doi:10.3390/genes14010209)
Supplement: Supplementary file 1 [file genes-14-00209-s001.zip › genes-2136746-supplementary.pdf]

Supplemental Data

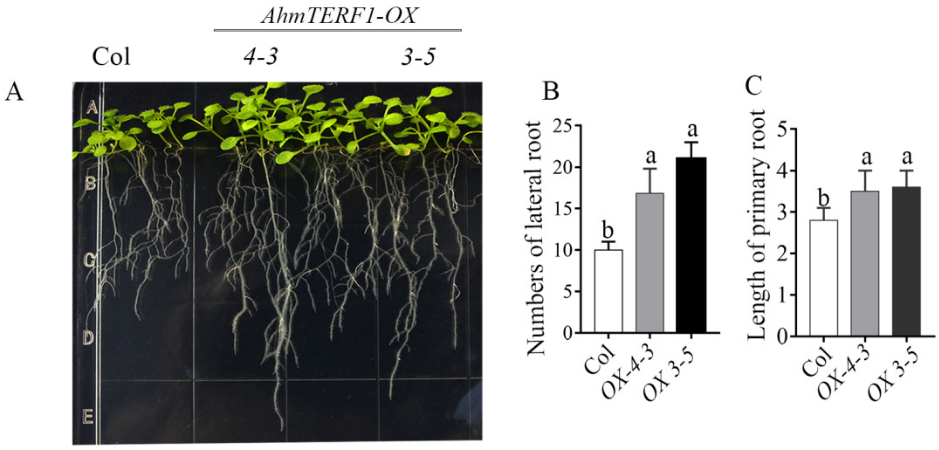

Supplemental Figure S1 *AhmTERF1* promotes root growth in *Arabidopsis*.

A: Phenotype of 7 d-old of Col and *AhmTERF1-OX Arabidopsis* lines grown on 1/2 MS medium. B and C: Number of lateral roots and length of primary roots of plants shown in A. Lower case letters (a, b) indicate significantly different groups (P<0.05).

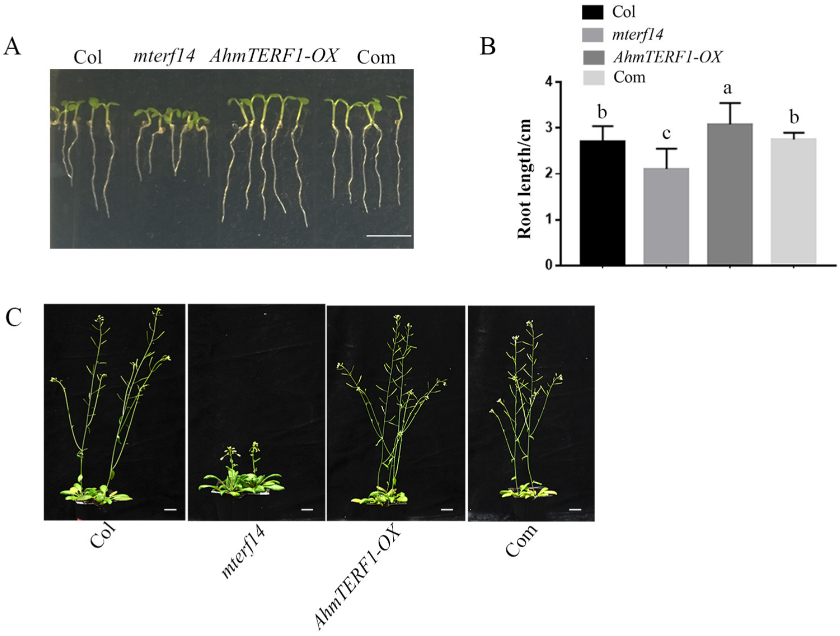

Supplemental Figure S2 *AhmTERF1* restores the phenotype of *mterf14* in *Arabidopsis.* A: Phenotype of 3 d-old *AhmTERF1* transgenic *Arabidopsis* seedlings grown on 1/2 MS medium. Scale bar: 1 cm. B: Length of roots from specimens in A. C: Phenotype of *AhmTERF1* transgenic *Arabidopsis* plants at the flowering stage. Scale bars: 1 cm. Lower case letters (a, b, c) indicate significantly different groups (P<0.05).

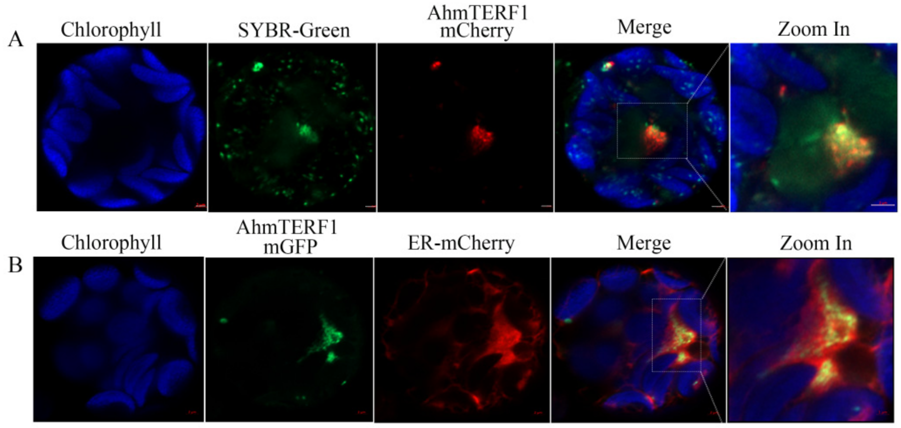

Supplemental Figure S3. AhmTERF1 co-localizes with mtDNA and ER.

A: AhmTERF1 co-localizes with mtDNA. B. AhmTERF1 co-localizes with ER.

Supplemental Table S1. Primers used in this study.

**Primer**	**Sequence**
AhmTERF1-qF	CATCCACACCACCATTTTCCTTCCA
AhmTERF1-qR	GACCCATTTGTGCCAGATTTTGCCT
AhmTERF1-GFP-F	CTGTACAAGCGGTACCCCGGGATGAAAACTTCATCTTCTCTTCATC
AhmTERF1-GFP-R	GTCCTAGGCTACGTAGGATCCTCAACTCGAATTCTTTTTCAG
pAhmTERF1-Gus-F	GATAAGCTTGATATCGAATTCTCTAGTTAAACTTATTTGGTCTGC
pAhmTERF1-Gus-R	AAGGGACTGTCTAGAACTAGTAGTTGTAGACTTGTAGTGGACCACA
Arahy.U6ZXMA -F	CAGTTGTTGTTCCACCTGACATTGA
Arahy.U6ZXMA -R	GCCAAGATGTCCTATTCTGAAAACC
atp9 -F	ATGTTAGAAGGTGCAAAATCAATGG
atp9-R	GAAAACAAAGAGAATCAGAAAGGCC

RRN18-F	AAGTTGGAATCGCTAGTAATCG
RRN18-R	TCCCCTACGGCTACCTTGTTACGAC
RRN26-F	AGACGAAAGTCGGCCATAGTGATCC
RRN26-R	TTCAACCCCAGGATGTGATGAGTCG
